# Genotypic variation in rice root exudates controls biological nitrification inhibition efficiency in the rhizosphere

**DOI:** 10.1093/ismejo/wrag254

**Published:** 2026-09-28

**Authors:** Jasmeet Kaur-Bhambra, Ellen Elizabeth Smith, Xiaoping Fan, Alaster Moffat, Joy Ebenezer Rajakulendran, Yiyu Meng, Marcel Jaspars, Cécile Gubry-Rangin

**Affiliations:** School of Biological Sciences, University of Aberdeen, Aberdeen, AB24 3UU, Scotland, United Kingdom; School of Biological Sciences, University of Aberdeen, Aberdeen, AB24 3UU, Scotland, United Kingdom; School of Biological Sciences, University of Aberdeen, Aberdeen, AB24 3UU, Scotland, United Kingdom; School of Biological Sciences, University of Aberdeen, Aberdeen, AB24 3UU, Scotland, United Kingdom; Marine Biodiscovery Centre, Department of Chemistry, School of Natural and Computing Sciences, University of Aberdeen, Aberdeen, AB24 3EU, Scotland, United Kingdom; School of Biological Sciences, University of Aberdeen, Aberdeen, AB24 3UU, Scotland, United Kingdom; Marine Biodiscovery Centre, Department of Chemistry, School of Natural and Computing Sciences, University of Aberdeen, Aberdeen, AB24 3EU, Scotland, United Kingdom; School of Biological Sciences, University of Aberdeen, Aberdeen, AB24 3UU, Scotland, United Kingdom

**Keywords:** nitrification, nature-based solutions, soil, BNI, nitrifier, plant genotype

## Abstract

Nitrification in agricultural soils drives significant nitrogen fertilizer losses and nitrous oxide (N_2_O) emissions, particularly in intensively fertilized rice systems. Biological nitrification inhibition (BNI), mediated by plant-derived compounds, offers a promising nature-based strategy to mitigate these losses, but the factors controlling BNI potential remain poorly understood. In this study, BNI mechanisms across five rice genotypes were investigated by combining plant physiology, soil nitrification dynamics, and exudate metabolomics. Nitrifier suppression was strongest in the rhizosphere and declined with distance from the root, indicating a spatially localized effect. BNI efficiency varied significantly among genotypes and selectively targeted nitrifiers with little effect on the broader microbial community. Ammonia-oxidizing archaea (AOA), the dominant nitrifiers in this acidic flooded soil, were more inhibited than ammonia-oxidizing bacteria (AOB) in soil and in exudate-amended culture assays. However, BNI preferentially altered AOB community diversity, whereas AOA diversity remained comparatively stable. Plant physiological traits, rather than the exudate metabolome, were more strongly associated with BNI efficiency. High-BNI genotypes exhibited greater shoot biomass, higher shoot N accumulation, and increased exudation, together with a higher relative abundance of *N*-butyldodecan-1-amine (NBDA) in their exudates. Metabolomic analyses identified a novel rice-derived BNI compound, NBDA, which strongly inhibited diverse nitrifier cultures, reduced soil N_2_O emissions, and showed no detectable off-target effects on non-nitrifying bacteria. Together, this study demonstrates that plant traits and the abundance of BNI-active compounds within the root exudate metabolome regulate BNI efficiency in the rhizosphere, advancing a predictive framework for mitigating nitrification in rice agroecosystems.

## Graphical abstract

**Figure:**
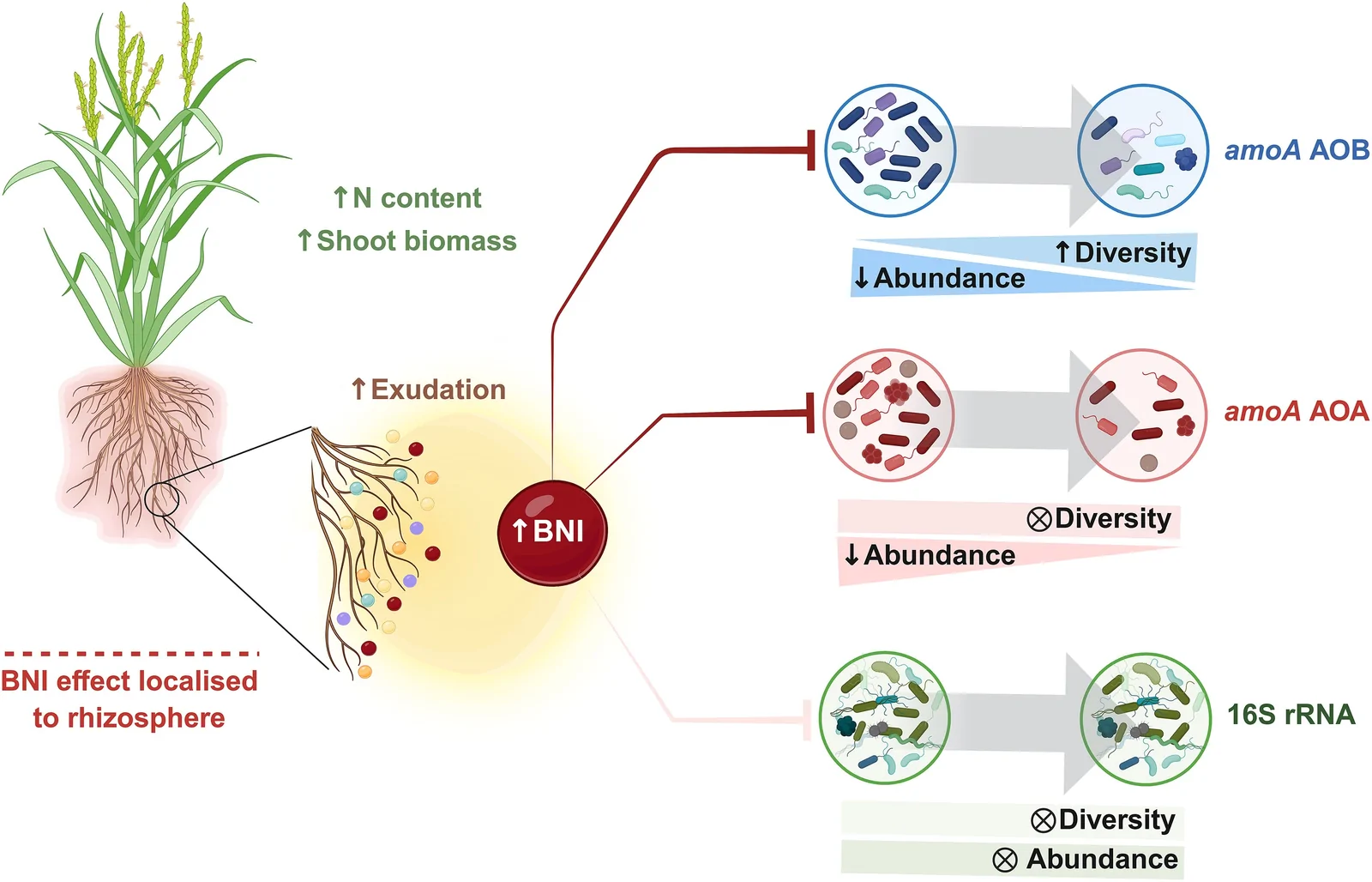

## Introduction

Agricultural soils use over 150 mT of nitrogen (N) fertilizer annually to sustain and increase crop yields [1, 2]. However, nitrification in agroecosystems results in substantial N losses, accounting for up to 50% of the applied fertilizers, and contributes to ~65% of global nitrous oxide (N_2_O) emissions [3, 4]. Rice (*Oryza sativa* L.), the staple food for half of the world’s population, consumes ~15% of the global N fertilizer, based on a global survey [1] and contributes towards ~0.18 Tg of N_2_O emissions annually [3]. Addressing the twin challenges of rising fertilizer demand and greenhouse gas emissions requires sustainable nitrification management strategies.

One promising nature-based approach is biological nitrification inhibition (BNI), in which some plant species release metabolites that suppress soil nitrifiers [5]. By reducing plant–nitrifier competition for N, BNI can improve plant nitrogen use efficiency (NUE), particularly in heavily fertilized cereals such as wheat [5], sorghum [6], maize [7], and rice [8]. Rice genotypes vary widely in NUE and rhizosphere nitrification activity [9, 10], with variation linked to differences in BNI capacity observed in both hydroponic and soil-based studies [8–12]. Two structurally distinct BNI compounds, a fatty alcohol, 1,9-decanediol [10], and a phenolic compound, syringic acid [11], have been previously isolated from rice root exudates and can act synergistically to suppress nitrification [11]. However, 1,9-decanediol accounts for only ~36% of rice BNI efficiency in the high-BNI genotype WYJ7, suggesting the presence of additional, unidentified compounds [10, 11].

Most of the known BNI compounds tested on a range of nitrifier cultures inhibit ammonia-oxidizing archaea (AOA) more efficiently than ammonia-oxidizing bacteria (AOB) [13–15]. Whether these patterns hold in soil remains unclear, as culture-based BNI efficiency frequently does not capture the edaphic complexity and in the presence of diverse microbial, viral, and eukaryotic interactions [7, 13–18]. Likewise, the broader effects of BNIs on nitrifier community structure and overall rhizosphere microbial diversity in rice ecosystems remain poorly understood [12], particularly because most BNI knowledge to date has been derived from controlled hydroponic systems. Therefore, a knowledge gap exists in linking BNI-associated chemistry to its ecological consequences *in situ*.

BNI efficiency in soil depends not only on inherent compound efficiency but also on its distribution in the soil matrix. Root rhizodeposition, including BNI compounds, can migrate beyond the rhizosphere, with the compounds’ chemical structures and polarities influencing their movement [19, 20]. For example, BNI compounds reported to date encompass a wide range of polarities, from hydrophilic to hydrophobic [10, 11, 21, 22]. In addition, their abiotic and biotic degradation in soil will also affect their efficiency. However, their diffusion, persistence, and inhibition efficiency gradients away from roots have yet to be quantified in soil systems. This spatial dimension is crucial, as exudate concentration typically declines sharply with distance from the root surface, potentially creating heterogeneous zones of nitrifier inhibition. Moreover, the rate and composition of root exudates are shaped by plant genotype, root functional traits, such as root diameter, tissue density, and nitrogen content, especially in forbs and grasses [23, 24].

Interdisciplinary advances in agronomy [5], plant genetics [25], biochemistry [26], microbiology [27], and pan-omics [28] have propelled BNI research; yet, few studies integrate plant physiology, metabolomics, and microbial ecology within a single soil framework. Such integration is necessary to provide a predictive understanding of BNI performance in soil. Here, using glasshouse-grown rice mesocosms, multiple factors controlling BNI efficiency *in situ* and their consequences on soil microbial communities were investigated. Interdisciplinary approaches, including physiology, microbiology, and chemistry, were applied to link exudate abundance and composition to nitrifier inhibition in soil. We tested three ecological hypotheses: H1, nitrifier inhibition is greater in the rhizosphere than in the extended rhizosphere, due to higher exudate concentrations; H2, BNI compounds inhibit AOA more efficiently than AOB in soil, similarly to bioassays in pure cultures; and H3, genotype-specific root architecture and exudate metabolome determine BNI efficiency.

## Materials and methods

### Rice mesocosm setup

Rice mesocosms were constructed using a pH 5.5 sandy loam agricultural soil. Five rice varieties, Lemont, IR64, Koshihikari, Azucena, and Moroberekan, were selected to represent a range of BNI efficiencies based on a previous study [8]. Each pot contained 1.5 kg of soil partitioned into two compartments by a mesh bag (2.5 μm mesh, Normesh, UK) enclosing 0.5 kg. A single seedling was transplanted in the centre of the mesh bag. The soil tightly adhering to the roots inside the mesh bag was designated as ‘rhizosphere’ soil, the soil outside the mesh bag within the pot as ‘extended rhizosphere’, and the soil from the unplanted pot as ‘bulk’ soil. Plants were grown under flooded conditions for 90 days. For each rice genotype and bulk soil control, triplicate pots were established at four sampling time points: 0, 30, 60, and 90 days (see supplementary methods). These time points corresponded to key vegetative/developmental stages on the BBCH scale: germination at Day 0 (BBCH 05), early tillering at Day 30 (BBCH 19–22), early stem elongation at Day 60 (BBCH 30–32), and late booting to heading at Day 90 (BBCH 43–52) [29].

At each sampling time point, triplicate pots were destructively harvested. The nylon mesh bag was carefully removed, and the rhizosphere soil was collected. Extended rhizosphere soil was sampled from the upper and lower 3 cm layers outside the mesh at the pot perimeter, with subsamples taken from three locations per pot and pooled. Bulk soil was collected from unplanted pots by coring the upper, middle, and lower 3 cm layers at five randomly selected positions per pot and pooling the subsamples. Plants were separated into root and shoot fractions, oven-dried at 60°C overnight, and weighed to determine dry biomass. Total nitrogen (%N) content of 90-day-old dried rice shoot samples was determined using an elemental analyser, with analytical quality control verified through replicate analyses of the certified reference material BCR-129 (hay powder). All soil samples were immediately frozen and stored at −80°C until molecular analyses.

### Quantification of microbial abundance and nitrifier inhibition in soil

AOA, AOB ammonia monooxygenase subunit A (*amoA*), and general 16S rRNA gene abundances were quantified using quantitative PCR (qPCR) from soil DNA extracts using primer pairs crenamoA23F/crenamoA616R [30], amoA-1F/amoA-2R [31, 32], and 515mF–806mR [33, 34], respectively (see supplementary methods). For each sampling time point, nitrifier abundance ratios and percentage inhibition of nitrifiers were calculated separately for AOA and AOB in each planted soil compartment (rhizosphere and extended rhizosphere):


(1) \begin{equation*} \mathrm{ Nitrifier }\ \mathrm{ abundance }\ \mathrm{ ratio } = \frac{\mathrm{gene}\ {\mathrm{copy}\ \mathrm{numbers}}_{planted\ compartment}}{\ \mathrm{gene}\ {\mathrm{copy}\ \mathrm{numbers}}_{bulk}} \end{equation*}

(2) \begin{equation*} \mathrm{ Inhibition } (\%) = (1 - \mathrm{ Nitrifier }\ \mathrm{ abundance }\ \mathrm{ ratio }) \times 100 \end{equation*}

To assess cumulative genotype-associated nitrifier suppression over 90 days, nitrifier abundance ratio values were natural-log transformed to improve data normality and satisfy model assumptions. Nitrifier ‘decline rates’ for each genotype were estimated as the slopes by fitting linear regressions to log-transformed nitrifier abundance ratio within the rhizosphere over time, and this was calculated independently for AOA and AOB. The resulting slopes representing decline rates were used as comparative proxies for ‘AOA BNI efficiency’ and ‘AOB BNI efficiency’ for AOA and AOB, respectively. Steeper slopes indicated stronger sustained nitrifier suppression. AOA and AOB decline rates were then summed to estimate the total nitrifier ‘BNI efficiency’ for each genotype.

Microbial abundance (*amoA* and 16S rRNA gene copies) and nitrifier inhibition data were natural-log transformed before statistical analyses to improve data normality and satisfy model assumptions. All conditions showing stimulation, as well as Day 0 data, were excluded from the statistical analyses of nitrifier inhibition to meet the assumptions of parametric analysis. Differences in microbial abundance (*amoA* and 16S rRNA gene copies), nitrifier inhibition, nitrifier decline rates, and spatial distribution of nitrifiers (rhizosphere versus extended rhizosphere) were analysed in R v4.2.1 using multifactor linear models and two-way ANOVA followed by post hoc comparisons where appropriate. Model parameters and associated statistical tests are provided in the SI tables.

### Microbial diversity

AOA *amoA*, AOB *amoA*, and 16S rRNA gene amplicons were amplified using primer pairs as mentioned above, with KAPA HiFi HotStart ReadyMix and sequenced using Illumina MiSeq V3 (2 × 300 bp) at Macrogen company. Raw reads were processed to amplicon sequence variants (ASVs) using the nf-core/ampliseq pipeline with custom parameters (see supplementary methods). Reads were rarefied to the minimum sample depth for each gene. The α- and β-diversity were analysed using phyloseq v1.55.0 [35]. Differences in β-diversity were assessed using Principal Coordinate Analysis (PCoA) of the Bray-Curtis dissimilarity distances between samples. Statistical associations between BNI activity and variation in α- and β-diversity were assessed by linear correlation models using Pearson correlation analyses for α-diversity and PERMANOVA and Mantel tests for β-diversity in R v4.3.0. AOA, AOB, and 16S rRNA gene diversity were correlated with AOA ‘BNI efficiency’, AOB ‘BNI efficiency’, and total ‘BNI efficiency’, respectively.

### Exudate collection and identification of BNI compounds

At Day 90, plants were removed from pots and roots were gently rinsed with deionized water to remove adhering soil particles. Plants were acclimated for 5 h in a sterile collection solution [36] (1 mM CaCl_2_ and 1 mM NH_4_Cl in deionized water) and subsequently transferred to darkened flasks containing 1 L of fresh collection solution for 36 h [37]. Due to plant uptake, the solution volume needed to be replenished to maintain the volume constant. To control for potential soil-derived carryover, 0.5 g of bulk soil was incubated in parallel in 1 L of collection solution as a medium-only control. Following incubation, exudate solutions were filter-sterilized (0.22 μm) and stored at 4°C for up to 6 days before downstream processing. Exudate solutions were filter-sterilized and concentrated by rotary evaporation at 40°C (see supplementary methods). Correlation analyses were used to assess relationships between the plant traits, exudate biomass, and total ‘BNI efficiency’.

Dried exudates were redissolved in 50% High-Performance Liquid Chromatography (HPLC)-grade methanol and analysed by liquid chromatography-mass spectrometry (LC-MS) in positive ionization mode using a Bruker MAXIS II mass spectrometer (Bruker Corporation, Billerica, MA, USA) equipped with a quadrupole-time-of-flight mass analyser coupled to an HPLC system (Agilent 1290 Infinity). Raw Bruker files were converted to mzML format using ProteoWizard and processed with PyOpenMS v3.3.0 for feature detection (2 ppm mass tolerance) and alignment (5 ppm mass tolerance) [38, 39]. Features were filtered based on blank-derived noise thresholds, chromatographic peak width (1–60 s), isotopic patterns, and median absolute deviation. Following batch-wise median normalization and log_2_ transformation, 881 features were retained for downstream analyses. Euclidean distance matrices were calculated and visualized by PCoA (see supplementary methods). Metabolite annotation was performed based on retention time and tandem mass spectrometry (MS/MS) fragmentation using Bruker DataAnalysis software and dereplication against the Reaxys database. Candidate peak of interest was further evaluated, and the presumed structure of the corresponding compound was confirmed by comparison with an authentic standard.

### Culture-based inhibitory effect of root exudates and newly isolated BNI compound

The nitrifier growth inhibition induced by root exudates from five rice genotypes and the newly identified pure BNI compound, *N*-butyldodecan-1-amine (NBDA) (see Results), was determined for three AOA (*Nitrososphaera viennensis* [40], *Nitrosotalea sinensis* [41], and *Candidatus* Nitrosocosmicus franklandianus [42]) and three AOB (*Nitrosospira tenuis* [43], *Nitrosomonas europaea* ATCC 19718, and *Nitrosospira multiformis* ATCC 25196). Root exudates were dosed at 2 mg mL^−1^ culture in 0.1% DMSO. The newly identified pure BNI compound was tested over the 0.2–50 μM range in the same solvent. Growth analyses and determination of inhibitory concentration at 80% inhibition (IC_80_) were performed as described previously [14] (see supplementary methods).

### Inhibitory effects of *N*-butyldodecan-1-amine on soil nitrification dynamics

A soil microcosm experiment was done to assess the dose-dependent effects of NBDA on nitrification and N_2_O emissions. Based on a previous study [16], NBDA treatments were applied at 0, 100, 500, 1000, and 1500 μg NBDA kg^−1^ dry soil, with three replicate microcosms per treatment. NBDA was dissolved in 2 ml of methanol, applied to 4 g of sand, dried, and then mixed with 16 g of soil to obtain an 80:20 soil-to-sand ratio in 100 ml ventilated polystyrene cups. The soil was adjusted to ~40% moisture content, and NH_4_Cl was added at 100 mg N kg^−1^ dry soil prior to incubation. Microcosms were incubated for 7 days, with ventilated lids. The short duration of the microcosm was considered due to the classical short life of BNIs in soil [16].

N_2_O emissions were measured daily using an Aeris analyser coupled to sealable gas chambers (6 cm diameter × 12 cm height; chamber volume 339 cm^3^). For each microcosm, gas concentrations were recorded continuously during 120 s chamber-closure periods and processed in the flux package in R v4.3.1. N_2_O fluxes were calculated from the linear increase in chamber headspace concentration over time, accounting for chamber volume, surface area, air temperature, and atmospheric pressure. Cumulative N_2_O emissions were estimated by trapezoidal integration of daily fluxes. At the end of the incubation, soils were destructively sampled for qPCR quantification of AOA *amoA*, AOB *amoA,* and bacterial 16S rRNA genes, and for nitrate and ammonium analyses (see supplementary methods).

## Results

### Taxon-specific ammonia oxidizer inhibition is favoured in the rhizosphere

Rice led to selective nitrifier inhibition rather than an overall decline in the soil microbial community. Total microbial abundance in the rhizosphere after 90 days differed among rice genotypes, with planted treatments generally showing increased, rather than reduced, microbial abundance relative to unplanted controls (Statistic S1; Fig. S1). Also, nitrifier abundance declined in bulk soil (Statistic S2; Fig. S2), likely due to the flooded conditions of the mesocosm. However, nitrifier inhibition was substantially greater in the planted compartments than in bulk soil (Statistic S3; Fig. S3), indicating a plant-mediated BNI effect on nitrifiers rather than a general inhibitory effect of the flooded experimental conditions.

Of the measured nitrifiers, AOA were the dominant nitrifiers in the slightly acidic soil (pH 5.5) and remained more abundant than AOB throughout the 90-day water-logged incubation (Figs S2 and S3), consistent with previous findings from acidic paddy soils [44, 45]. Across all genotypes, the nitrifiers (AOA and AOB) were much more inhibited in the rhizosphere than in the extended rhizosphere (Statistic S4; Fig. 1), supporting a stronger inhibition closer to the root than further away (H1). This effect was not influenced by the mesh experimental setup (Fig. S2).

**Figure 1:**
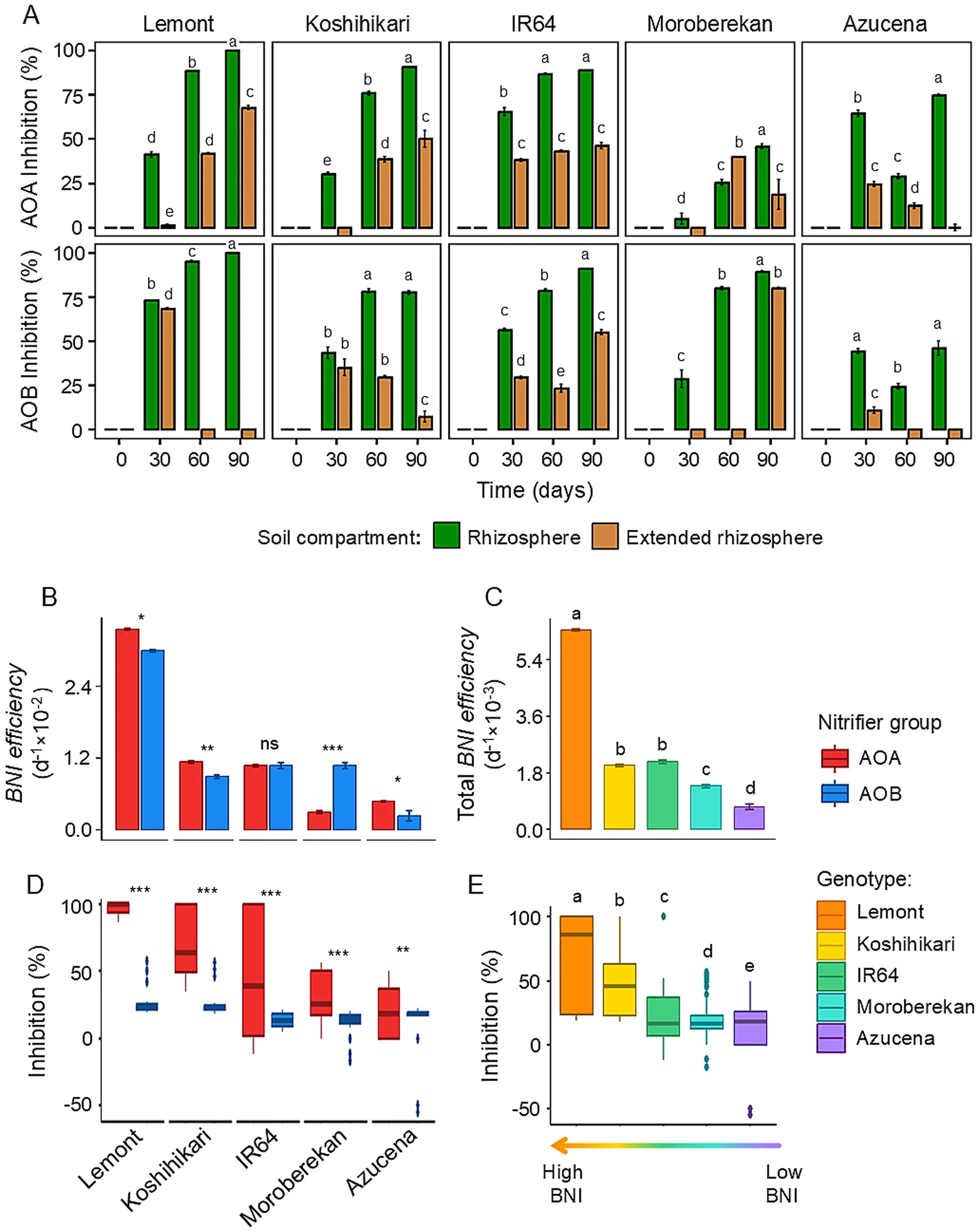
Rice genotype-driven spatial and temporal patterns of nitrifier inhibition and BNI efficiency. (A) Inhibition of AOA and AOB abundance in the rhizosphere and extended rhizosphere relative to bulk soil over time for the five rice genotypes. (B) The ‘BNI efficiency’ of AOA and AOB in the rhizosphere of the five rice genotypes. The ‘BNI efficiency’ reflects the decline rates were estimated from linear temporal changes in nitrifier abundances relative to bulk soil over 90 days in the rhizosphere. (C) The total ‘BNI efficiency’ in the rhizosphere of the five rice genotypes was estimated as the total decline rate of nitrifiers (AOA + AOB). (D) Inhibition of AOA and AOB growth rates in culture bioassays amended with root exudates from the five rice genotypes. (E) Overall exudate-mediated inhibition of rice genotypes estimated by integrating growth inhibition across six ammonia-oxidizing strains; three AOA strains—*N. viennensis*, *N. sinensis,* and Ca. N. franklandianus—and three AOB strains—*N. europaea, N. multiformis,* and *N. tenuis*. Data are shown as bar plots with standard errors (*n* = 3) for A–C and as boxplots (*n* = 3) for D and E. Different letters indicate significant differences among genotypes (*P* < .01). Statistical comparisons between AOA and AOB are indicated above each pair, where ^***^ denotes *P* < .001, ^**^ denotes *P* < .01, ^*^ denotes *P* < .05, and ns denotes *P* > .05.

Inhibition of nitrifier abundance in the rhizosphere progressively increased over the 90-day growth period, with the strongest inhibition observed at Day 90. To capture the cumulative BNI effect across the experiment, rather than at a single time point, the nitrifier decline rate was estimated as the rate of proportional decline in rhizosphere nitrifier abundance relative to bulk soil (Fig. S4). This value was used as a proxy for ‘BNI efficiency’ for each rice genotype and is referred to as such throughout the manuscript. For three of the five rice genotypes, AOA ‘BNI efficiency’ was greater than AOB ‘BNI efficiency’, whereas the opposite pattern was observed for IR64 and Moroberekan (Statistic S5; Figs 1B and S5), suggesting that under the acidic conditions tested, the BNI compounds may inhibit AOA more efficiently than AOB not only in cultures, but also in soil, but with low confidence (H2). The total ‘BNI efficiency’ for the five rice genotypes was then inferred using the sum of AOA and AOB decline rates, classifying them on a spectrum of ‘BNI efficiency’ from high- to low-BNI genotypes as follows: Lemont, IR64, Koshihikari, Moroberekan, and Azucena (Statistic S6; Fig. 1C).

The BNI activity of the different rice genotypes was also evaluated by measuring the inhibition of AOA and AOB cultures in the presence of root exudates collected from the same plants after 90 days of growth. Similarly to soil, AOA cultures were more inhibited by the root exudates than their AOB counterparts (supporting H2), with strain-specific responses (Statistic S7; Figs 1D and S6). Such strain-specific responses suggest that bioassays using a single strain may fail to capture the complexities of soil BNI response. However, the inclusion of multiple AOA and AOB strains into a unique BNI activity estimate (Fig. 1E) not only demonstrates a rice genotype effect on BNI activity but also ranks the variability in rice genotype BNI activity similarly to that in soils (Fig. 1C and E). Standardizing culture bioassays to an equal total exudate dose revealed both qualitative and quantitative differences among genotypes. Exudates from high-BNI genotypes exhibited greater inhibitory activity per unit exudate, indicating a higher relative abundance or better efficacy of active BNI compounds.

Soil ammonium and nitrate dynamics were assessed as complementary indicators of BNI efficiency (Fig. S7). Ammonium consumption was lower in soils planted with high-BNI genotypes than in those with low-BNI genotypes, although it remained substantially lower than in the unplanted control. Nitrate was not detected in the mesocosms after the start of the experiment, likely due to leaching under flooded conditions. However, because rice can assimilate both ammonium and nitrate and also influence nitrifier abundance, these measurements cannot directly disentangle nitrification inhibition from plant nitrogen uptake.

### BNI efficiency selectively restructures AOB, but not AOA, communities

The microbial sequencing depth applied (Fig. S8) ensured robust coverage of microbial diversity across all samples (Fig. S9). *Nitrosospira* were the dominant AOB in the soil, with acidic soil-associated D12, D15, D16, and D19 clusters being the dominant clades (Fig. S10) [46]. Rice genotype and the corresponding AOB ‘BNI efficiency’ significantly shaped AOB α- and β-diversity within the rhizosphere (Statistic S8; Figs S11 and S12). These effects also extended to the extended rhizosphere, where α- and β-diversity were similar to those in the rhizosphere, suggesting that AOB BNI effects were not confined to the immediate root zone (Statistic S9; Fig. S13). AOB ‘BNI efficiency’ was positively correlated with AOB diversity, including Inverse Simpson (Fig. 2A) and community dissimilarity (Fig. 2B), suggesting that BNI activity preferentially suppresses dominant AOB taxa, with limited impact on rare ones.

**Figure 2:**
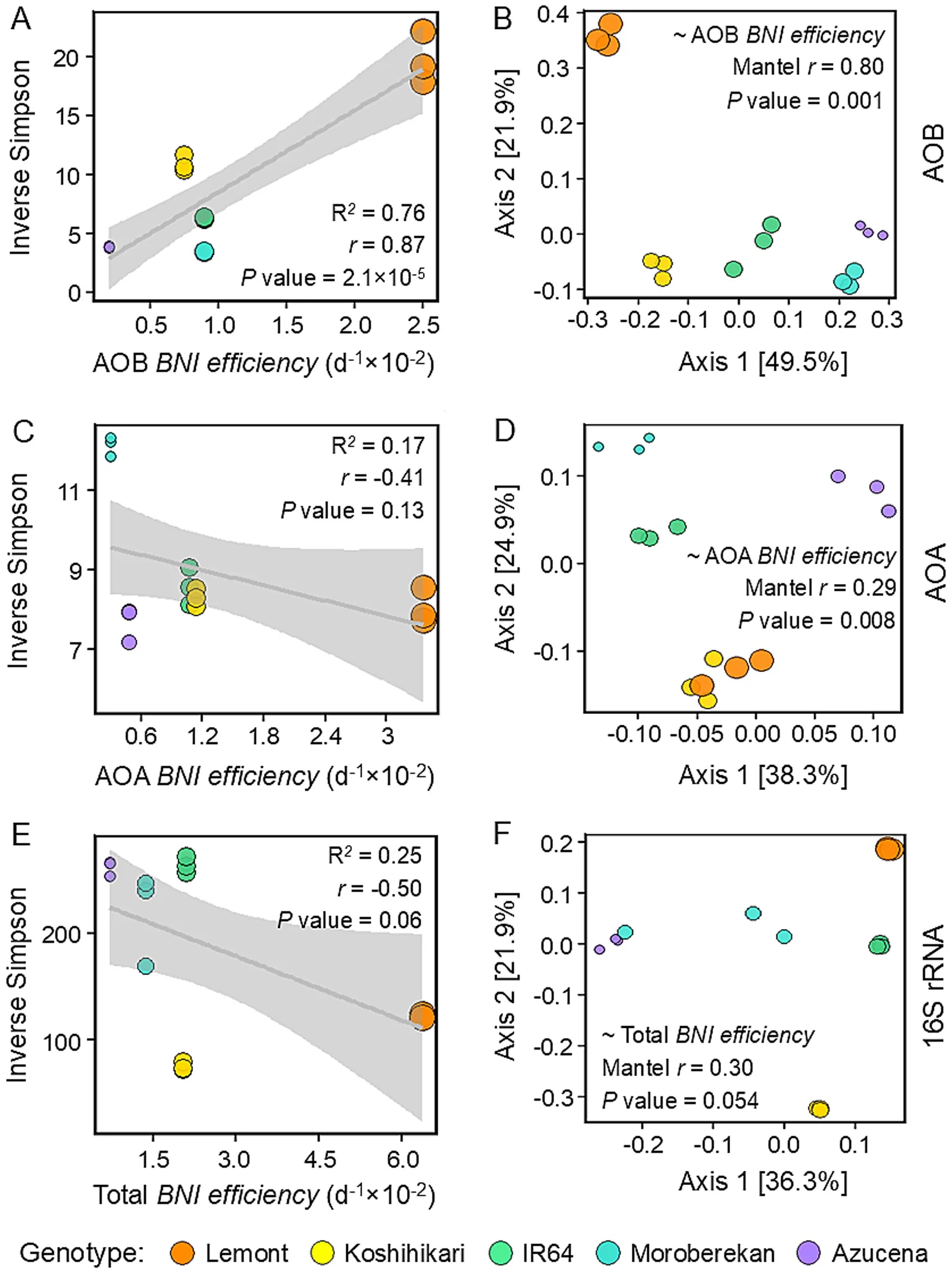
Impact of rice BNI efficiency on soil microbial community structure and diversity. Correlations between AOB ‘BNI efficiency’ and AOB α-diversity (inverse Simpson index; (A) and β-diversity (Bray-Curtis dissimilarity; (B) equivalent analyses were performed for AOA ‘BNI efficiency’ and AOA α-diversity (C) and β-diversity (D), and for total ‘BNI efficiency’ with total bacterial α-diversity (E) and β-diversity (F). Each point represents an individual sample. Points are sized according to ‘BNI efficiency’. For α-diversity regressions, shaded areas indicate 95% confidence intervals, with R*^2^*, Pearson correlation coefficients (*r*), and *P*-values reported within each panel. For β-diversity PCoA plots, Mantel correlation coefficients (*r*) and *P*-values are reported within each panel.

Nitrososphaera NS-γ [Cluster C11], a clade commonly associated with acidic soils, dominated the AOA community throughout the experiment, followed by NS-δ [Cluster C1], with both clades remaining largely stable across rice genotypes and soil compartments (Fig. S10) [47]. AOA α- and β-diversity differed significantly among rice genotypes and between the rhizosphere and extended rhizosphere within each genotype (Statistic S10; Figs S11 and S12). However, the lack of correlation between compartments suggests compartment-specific structuring of AOA communities, with plant-associated effects largely confined to the immediate rhizosphere (Fig. S13). Within the rhizosphere, AOA ‘BNI efficiency’ did not correlate with AOA α-diversity (Fig. 2C) and had only a weak correlation with community composition (Fig. 2D), indicating that additional factors beyond BNI contribute to AOA community assembly. An observed correlation between AOA and AOB diversity is consistent with, but does not directly demonstrate, potential competitive interactions between these nitrifier groups (Fig. S13).

Chloroflexota, Pseudomonadota, and Actinobacteriota were the most abundant bacterial phyla across all samples (Fig. S14). Within the rhizosphere, the relative abundance of the AOA-affiliated order *Nitrososphaerales* (phylum Thermoproteota) decreased with increasing total ‘BNI efficiency’ across rice genotypes (Fig. S14). Similar to AOA, total bacterial α- and β-diversity differed significantly among rice genotypes and between the rhizosphere and extended rhizosphere within each genotype (Statistic S11; Figs S11 and S12). Within the rhizosphere, total ‘BNI efficiency’ did not significantly correlate with changes in total bacterial α- or β-diversity (Fig. 2E and F), suggesting that BNI has a minimal effect on the overall soil microbial community. Also, as AOA and AOB are components of the broader microbial community, their suppression may contribute to some of the observed community-level differences. The variation in total bacterial diversity was associated with nitrifier diversity, showing a stronger relationship with AOB diversity and a weaker correlation to AOA diversity (Fig. S13).

### Plant physiology drives BNI efficiency

Rice plants with greater total ‘BNI efficiency’ showed higher shoot N content (Statistic S12; Fig. 3A and B). In addition, shoot biomass and root exudation mass were positively correlated with total ‘BNI efficiency’. In contrast, root biomass was negatively correlated (Figs 3B and S15), indicating that high-BNI genotypes tend to allocate more biomass above ground, accumulate more N, and release greater quantities of root exudates. Together, these findings support a link between plant physiological traits and total ‘BNI efficiency’ (H3). Metabolomic profiling of high-molecular-weight root exudates (Fig. S16) revealed pronounced genotype effects, with Koshihikari dominating exudate compositional variation, followed by Lemont, with the remaining genotypes contributing relatively little to the overall variance (Figs 3C and S17).

**Figure 3:**
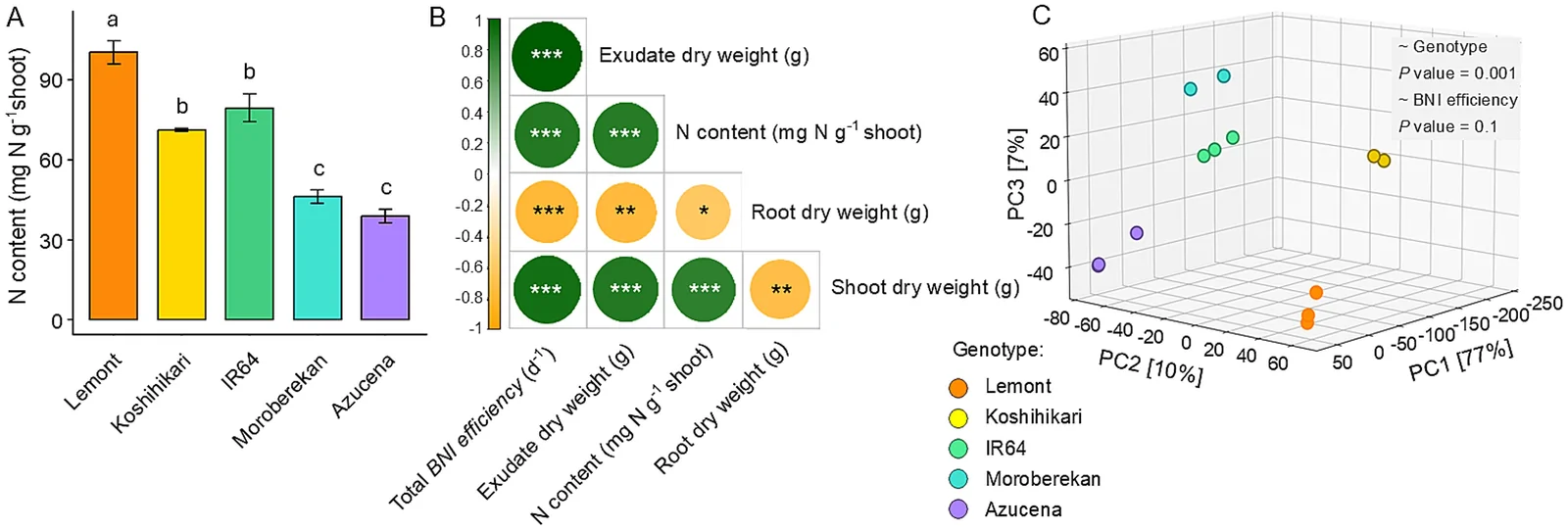
Influence of plant physiological and exudation traits on rice 'BNI efficiency'. (A) Shoot N content of the five rice genotypes (Lemont, Koshihikari, IR64, Moroberekan, and Azucena). Data are presented as a bar plot, and standard errors (*n* = 3) are indicated by error bars. Different letters on top of each bar denote significant differences (*P*-value < .05), tested using ANOVA, followed by Tukey-adjusted post hoc comparisons (B) Pearson correlation matrix showing relationships among ‘BNI efficiency’ and plant physiological traits, including exudate dry weight, shoot N content, root dry weight, and shoot dry weight. Circle colour and size represent the direction and strength of correlations, respectively. Symbols ^*^, ^**^, and ^***^ denote statistical significance at *P* < .05, *P* < .01, and *P* < .001, respectively. (C) Three-dimensional principal component analysis (PCA) of high-molecular-weight root exudate composition from 90-day-old rice plants, based on LC-MS profiles (*n* = 3 per genotype). PERMANOVA *P*-values showing the relationships between exudate metabolome composition, rice genotype, and ‘BNI efficiency’ are reported within the panel.

The previously reported rice BNI compounds 1,9-decanediol and syringic acid could not be detected in our LC-MS analyses, as they fall outside the higher m/z detection range targeted in this study. Using targeted natural product discovery at higher m/z, we identified a prominent LC-MS peak at 3 min (m/z 242.28), corresponding to [M^+^H]^+^ of C_16_H_36_N and assigned as NBDA (Figs 4A and S18). MS/MS fragmentation matched published spectra and an authentic standard [48], confirming NBDA as a linear aliphatic amine and a previously unreported class of BNI compound (Fig. S18). NBDA was detected in all genotypes except the low-BNI line Azucena, with higher signal intensity in high-BNI genotypes, closely matching their measured BNI effect (Figs 4B and S19).

**Figure 4:**
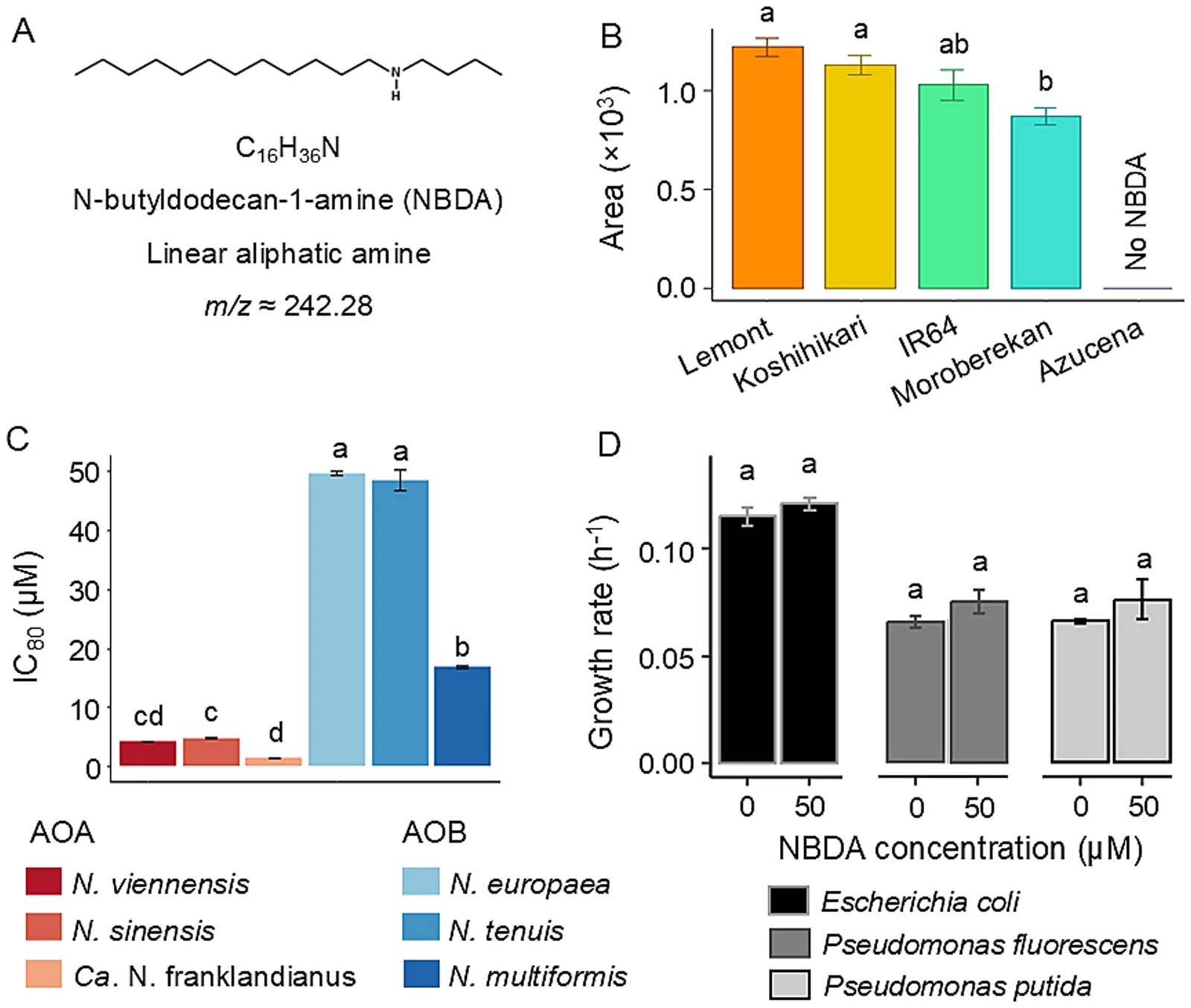
Identification and efficiency of NBDA, a newly identified rice biological nitrification inhibitor. (A) The molecular structure of the newly identified rice BNI compound NBDA. (B) NBDA signal of peak area in Day 90 exudates across the five rice genotypes. Azucena showed no peak for NBDA. Data are presented as a bar plot, and standard errors (*n* = 3) are indicated by error bars. (C) Inhibitory concentrations at 80% inhibition (IC_80_) of six ammonia-oxidizer strains for NBDA. Three AOA strains—*N. viennensis*, *N. sinensis,* and Ca. N. franklandianus—and three AOB strains—*N. europaea, N. multiformis,* and *N. tenuis*—were used in this assay. (D) Comparison of growth rates of three non-nitrifier strains, *Escherichia coli* MG1655, *Pseudomonas fluorescens* NCIMB 10586, and *Pseudomonas putida* PG NCIMB 9816, in the absence and presence of 50 μM of NBDA. Data are presented as a bar plot, and standard errors (*n* = 4) are indicated by error bars. Different letters on top of each bar denote significant differences (*P*-value < .05) in IC_80_ or growth rate.

### NBDA is a highly effective BNI compound

NBDA exhibited strong inhibitory activity, with IC_80_ values ranging from 1.3 to 49.8 μM across the six ammonia-oxidizing strains tested (Statistic S13; Fig. 4C). Despite its structural similarity to 1,9-decanediol, NBDA was ~30-fold more potent than this previously reported rice BNI compound [11, 14] (Table S1). NBDA inhibited AOA more strongly than AOB (Fig. 4C), consistent with responses reported for other BNI compounds tested using pure cultures [14]. Hydroxylamine supplementation did not rescue growth in either AOA or AOB under NBDA exposure (Fig. S20), indicating that NBDA likely targets an enzymatic step distinct from the canonical ammonia oxidation pathway. NBDA did not inhibit three common non-nitrifying terrestrial strains at concentrations up to 50 μM (Figs 4D and S21), suggesting the absence of off-target effects.

NBDA activity was further tested in soil microcosms established under the same soil and moisture conditions as those for the plant mesocosms. NBDA reduced ammonium consumption in a dose-dependent manner (Statistic S14; Fig. 5A), with a stronger effect on N_2_O emissions, decreasing fluxes by ~40% at 100 μg g^−1^ soil and up to ~80% at 1500 μg g^−1^ soil (Figs 5B and S22). This reduction occurred from the start of incubation (Fig. S22) and was accompanied by reduced AOB abundance, whereas AOA abundance was unaffected, likely due to the short incubation period and N fertilization (Statistic S15; Figs 5C and S23). N_2_O flux was strongly correlated with AOB abundance, indicating that emission reductions were linked to AOB suppression (Fig. 5D). NBDA had no detectable effect on total bacterial abundance, supporting limited off-target effects (Fig. S23).

**Figure 5:**
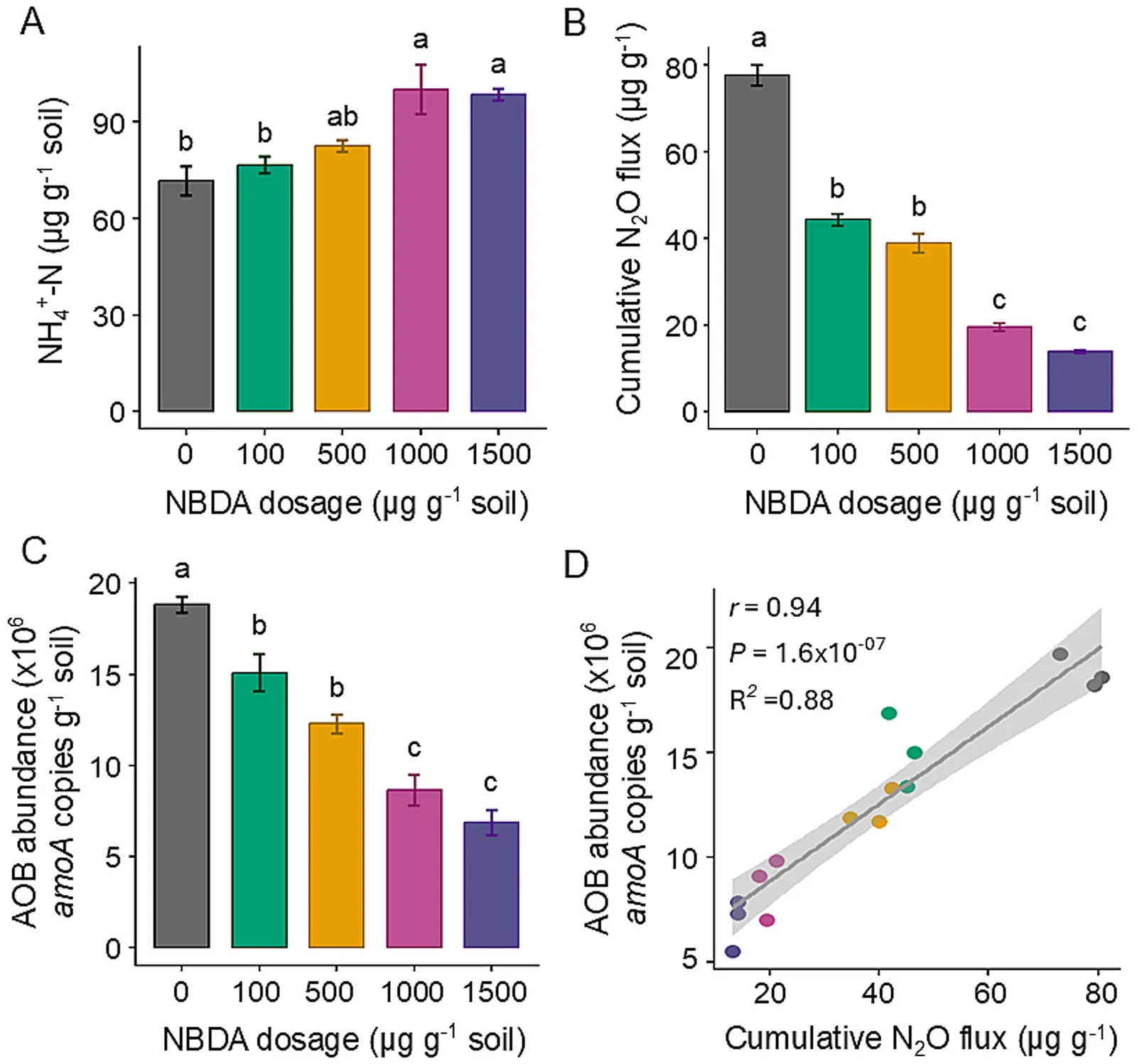
Effects of NBDA on soil N dynamics, nitrifier abundance, and N_2_O emissions in soil microcosms. Ammonium-derived soil mineral N content (A), cumulative N_2_O emissions (B), and AOB abundance (C) following 7 days of incubation of a pH 5.5 soil amended with increasing NBDA concentrations (0, 100, 500, 1000, and 1500 μg g^−1^ soil). Data are presented with bar plot, and standard errors (*n* = 3) are indicated by error bars. Different letters on top of each bar denote significant differences among treatments (*P*-value < .05). (D) Correlation between cumulative N_2_O emissions and AOB abundance across NBDA treatments. In the regression analysis, the shaded area represents the 95% confidence interval, with R*^2^*, Pearson correlation coefficient (*r*), and *P*-values reported within the panel.

## Discussion

### Spatial localization of BNI compounds in soil

Root exudates form radial concentration gradients, with the highest concentrations at the root surface and declining with distance. The extent of this gradient is shaped by compound chemistry, such as polarity, as hydrophilic compounds disperse farther through the soil pores, whereas hydrophobic compounds remain proximal to the roots [19–22, 49]. Although flooded rice mesocosms favour solute transport through continuous water-filled pores [49, 50], in our study, the BNI effect was largely confined to the rhizosphere, with little effect in the extended rhizosphere, supporting H1 (Fig. 1). This localization aligns with the chemistry of rice-derived BNIs, NBDA (this study) and 1,9-decanediol [10] being hydrophobic, as are other detected key rice allelochemicals, such as momilactones, also hydrophobic in nature [51, 52]. In water-logged paddy soils, hydrophobic inhibitors are advantageous because they resist leaching and maintain effective concentrations at the root-soil interface [19–22, 49]. Moreover, the acidic soil pH in our system (<5.5) would increase sorption of phenolic compounds to mineral and organic surfaces [53, 54], further limiting exudate mobility.

Many studies showing a higher abundance of hydrophilic over hydrophobic BNIs used hydroponic-based settings [21, 22, 55]. Different growth systems (soil vs. sterile hydroponic) alter exudation via changes in root physiology, soil structure and microbial-root interactions, shifting exudate polarity and composition [19, 50, 56–60]. This likely explains why the BNI activity of our genotypes in soil deviated from prior hydroponic observations [8]. Indeed, soil-hydroponic comparisons indicate that soil-grown plants can exhibit a higher proportion of hydrophobic exudates [60]. Together, these observations suggest that compound polarity, soil pH/sorption, and growth environment jointly determine the spatial footprint of BNI activity.

### Ecology of nitrifier-specific inhibition of BNI

AOA generally were more sensitive to inhibition than AOB across the soil mesocosms, exudate bioassays, and NBDA culture assays, providing some support for H2. However, given the variability among rice genotypes and experimental systems, this trend should be interpreted with caution and not assumed to apply broadly across all soils, plant genotypes, or BNI compounds (Figs 1 and 4). This pattern may reflect two complementary mechanisms. First, AOA were the dominant nitrifiers in our acidic, low-ammonium soil system and low-oxygen-concentration environments [47, 61–65]. Several BNI studies have found that the most abundant ammonia-oxidizing clade in soil is often the one most strongly affected by inhibition, likely because its greater numerical abundance increases its overall exposure to, and interactions with, BNI compounds released into the soil [17, 66–69]. Second, AOA may be more sensitive to rice-derived BNIs than AOB [14, 15], leading to stronger abundance-based (rather than activity-based) inhibition. However, this interpretation should be approached with caution, as prior evidence for AOA sensitivity is based on cultured representatives that do not reflect the dominant AOA lineages in our system. Because these dominant clades currently lack cultured representatives, their specific sensitivity to BNIs cannot be determined definitively. In addition to direct BNI effects, nitrifier dynamics in flooded rhizosphere soils may also be influenced by rhizosphere acidification, oxygen availability, carbon release through root exudation, and competition with stimulated heterotrophic communities [20, 27, 70–72]. Thus, the stronger suppression of nitrifiers likely reflects direct BNI effects but may also be influenced by broader rhizosphere-mediated processes. Further mechanistic insight into genotype-dependent BNI will require integrating stable isotope approaches, particularly ^15^N-isotopic tracing, to quantify nitrogen transformation pathways in the rhizosphere, providing a direct functional link between BNI activity and nitrogen cycling, complementing the abundance- and community-based analyses presented here.

Despite stronger inhibition than AOB, the AOA community structure remained relatively stable over time, possibly due to broader stress tolerance. Similar stability of AOA community composition under BNI exposure has been observed previously for other plant-derived BNIs [16, 73]. In contrast, greater inter-strain variability in BNI sensitivity among AOB [14] likely drove cumulative changes in diversity and gradual community restructuring under sustained inhibitory pressure, as reflected by increased Simpson diversity under high inhibition (Fig. 2). Together, these results suggest that, in our study, AOA are sensitive to BNIs in terms of abundance but their communities buffer diversity under sustained inhibition efficiency, whereas BNIs impose stronger long-term selective pressure on AOB. However, the generality of this observation remains to be established, as a few major clades dominated ammonia oxidizer diversity in our study, and responses may differ for other AOA or AOB lineages under different soil conditions, plant genotypes, or time frames. In addition, comammox *Nitrospira*, which were not assessed in this study, are increasingly recognized as important contributors to nitrification in agricultural soils and may exhibit distinct responses to BNIs [74]. Extending the investigation of BNI effects from AOA and AOB to include comammox will therefore be an important direction for future research, providing a more comprehensive understanding of how BNIs influence the entire ammonia-oxidizing community. The limited effects of BNI on overall microbial diversity in this study further indicate that BNIs may act with taxonomic specificity rather than as broad-spectrum suppressors [68, 75].

### Key influence of plant physiological traits on BNI efficiency

Genotype-driven variation in plant physiology is known to shape root exudation patterns and rhizosphere microbial assembly [23, 24, 28, 49]. Our findings support this view and further demonstrate a close association between plant physiological traits and BNI efficiency (H3). In our system, phenotypic variation among rice cultivars explained more variation in ‘BNI efficiency’ than the exudate metabolome alone. High-BNI genotypes showed greater shoot biomass, higher shoot N accumulation, and elevated exudation rates, together with a higher relative abundance of BNI compounds in their exudates (Figs 3 and 4). By suppressing nitrification, these genotypes may retain more soil N as ammonium, which is generally less prone to leaching and denitrification losses than nitrate under flooded conditions [76]. Consistent with this, shoot biomass and shoot N content, both commonly used as indicators of nitrogen-use efficiency [77–79], and ammonium-use efficiency, previously linked to rice BNI capacity [8], were positively associated with ‘BNI efficiency’, suggesting that high-BNI genotypes convert available N into aboveground growth more effectively.

High-BNI genotypes also exhibited reduced root biomass but increased exudation, consistent with a compensatory chemical foraging strategy in which plants invest more carbon into rhizosphere exudation rather than root expansion [23, 24, 80, 81]. However, because plants were grown in mesh bags, root development may have been partially constrained, possibly reducing root biomass. This experimental setup may therefore have influenced the observed differences in root biomass, as well as patterns of root exudation and possibly the associated microbiome, and should be considered when interpreting these findings. Root exudation may also indirectly influence BNI efficiency by modifying rhizosphere physicochemical conditions, including available carbon, pH, redox status, nutrient availability, and microbial activity. Therefore, the observed relationships should be interpreted within a broader plant–soil–microbe framework, rather than as direct effects of BNI compounds alone [20, 70–72, 82]. Together, these findings suggest that genotype-specific life-history strategies may contribute to variation in BNI potential. Because plant physiology and metabolite production are dynamic, future studies should evaluate these relationships across developmental stages, environments, and soil–microbiome interaction contexts [57, 59, 81, 82].

### Rice-BNI NBDA demonstrates selective and soil-active BNI function

NBDA is a linear aliphatic amine and represents a previously unreported class of plant-derived BNI compounds. Although its precise mode of action was not resolved, high-molecular-weight amines can exert allelopathic effects through cationic detergent-like disruption of membrane-associated processes, including Na^+^/K^+^-ATPase activity and protein-phospholipid interactions [83]. Consistent with this possibility, low-molecular-weight alkyl amines, such as *n*-propylamine, have previously been shown to inhibit nitrifiers in wastewater systems [84]. NBDA inhibited AOA more strongly than AOB in culture, yet only AOB suppression was detected in soil. The absence of AOA inhibition in soil likely reflects their absence of growth; indeed, the high ammonium input and short incubation period favour rapid AOB growth and activity, hence outcompeting AOA [63–67]. The strong correlation between AOB abundance and N_2_O flux confirms the strong contribution of AOB to N_2_O emissions, as found in many soil studies [85–87], although other processes may also have contributed [88]. NBDA had no detectable effect on total bacterial abundance in either culture assays or soil, supporting limited off-target effects and aligning with observations from the rice mesocosms. Because soil physicochemical properties strongly influence nitrification inhibitor efficacy by affecting compound mobility, persistence, and degradation [16, 22], future work should test NBDA activity across contrasting soil pH, moisture, and redox conditions.

To our knowledge, this study provides the first step towards resolving the spatial localization of BNIs in soil, demonstrating that even under flooded conditions, effective BNI action remains mostly restricted to the rhizosphere. The BNI efficiency of rice increased with time and was more efficient towards dominant nitrifiers, which is partially explained by the nitrifier community composition and diversity. We identified that plant physiology in terms of N content, biomass, root exudate release, and composition contributes towards a plant’s BNI efficiency. We also reported that NBDA is a new potent nitrifier-specific inhibitor, capable of reducing nitrous oxide emissions in soil, expanding the known chemical diversity of plant-derived BNI compounds. Further detailed mechanistic resolution will require ^15^N-isotopic tracing to quantify nitrogen fluxes, providing a direct link between BNI activity and nitrogen transformation rates, alongside taxon-specific activity measurements, inclusion of other key nitrifying taxa such as comammox and nitrite-oxidizing bacteria, and analyses of BNI fate in soil.

## Supplementary Material

Supplementary_materials_wrag254

## Data Availability

All sequencing data have been deposited under the BioProject number PRJNA1238847 (https://dataview.ncbi.nlm.nih.gov/object/PRJNA1238847?reviewer=i491ojams20a15967d9pgsrlrd). All data scripts are available at: https://github.com/jasmeetkaur-bhambra/Rice-BNI.2022.
