# Supplementary material for: Genotypic variation in rice root exudates controls biological nitrification inhibition efficiency in the rhizosphere
Source: ISME J. 2026 Sep 28;20(1):wrag254. doi: 10.1093/ismejo/wrag254 (PMC13688362; doi:10.1093/ismejo/wrag254)
Supplement: Supplementary file 1 [file supplementary_materials_wrag254.zip › Supplementary_final_jkb_wrag254.docx]

**Supplementary data**

| Method | 2 |
| --- | --- |
| Supplementary Figures |  |
| Figure S1: Total microbial abundance in soil. | 7 |
| Figure S2: Decline in nitrifier abundance in bulk soil. | 8 |
| Figure S3: Abundance of nitrifiers in soil. | 9 |
| Figure S4: The ratio of abundance of nitrifiers in the soil compartments over time. | 10 |
| Figure S5: The abundance of nitrifiers in the bulk soil. | 11 |
| Figure S6: Biological nitrification inhibition of nitrifiers in culture by rice root exudates. | 12 |
| Figure S7: Soil ammonium and nitrate dynamics in rice rhizosphere over time. | 13 |
| Figure S8: Sequencing depth | 14 |
| Figure S9: Rarefaction curves | 15 |
| Figure S10: Ammonia oxidiser community composition. | 16 |
| Figure S11: Alpha diversity. | 17 |
| Figure S12: PCoA plot of beta diversity. | 18 |
| Figure S13: Conceptual correlation network. | 19 |
| Figure S14: Microbial community composition plots. | 21 |
| Figure S15: Correlation between plant traits and BNI efficiency. | 22 |
| Figure S16: Density distribution of mass-to-charge (m/z) values detected in the LC-MS analysis of all the rice root exudates. | 23 |
| Figure S17: PCoA of rice metabolome. | 24 |
| Figure S18: Identification of rice BNI, N-butyldodecan-1-amine (NBDA). | 25 |
| Figure S19: Relative quantification of NBDA in rice root exudates by LC–MS. | 26 |
| Figure S20: Inhibition mechanism of rice BNI, NBDA. | 27 |
| Figure S21: Inhibitory effects of NBDA on non-nitrifying microorganisms. | 28 |
| Figure S22: Inhibitory effects of NBDA dosage on soil nitrification dynamics. | 29 |
| Figure S23: Inhibitory effects of NBDA dosage on soil microbial dynamics. | 30 |
| Supplementary Tables |  |
| Table S1: Reported inhibitory concentrations of BNI compounds against nitrifiers. | 31 |
| Supplementary References | 34 |
|  |  |
|  |  |

**Methods**

**Soil sampling**

A sandy loam agricultural soil of pH 5.5 was sampled on 28 April 2021 from the upper 10 cm of a long-term pH-maintained plot in SRUC, Craibstone, Scotland (grid reference NJ872104). Soil characteristics are as described previously [1]. The soil was dried overnight at 40°C, sieved with a 3.35 mm mesh size and stored at 4°C before the construction of mesocosms.

**Seed Germination**

Seeds of rice varieties were sterilised with 5% H_2_O_2_ for 15 min, rinsed and soaked with deionised water for 2 h. The seeds were germinated on wet filter paper in Petri dishes at 40°C in the dark. After 4 days of incubation, the seeds were placed under light till shoots reached a length of about 1 cm.

**Mesocosm set up**

Mesocosms were prepared in black polyethylene containers (height, 22 cm; diameter, 17 cm) with 1.5 kg of dry soil equivalent per mesocosm. KCl, Na_2_HPO_4_ and NH_4_Cl were applied as basal fertilisers at 17 mg K, 50 mg P and 100 mg N per kg of dry soil. Nylon mesh bags (2.5 µm mesh, 18 cm height; 15 cm diameter) (obtained from Normesh, UK) containing 0.5 kg of dry soil equivalent were placed into the centre of each pot containing 1 kg of dry soil equivalent, creating two independent soil compartments through which water, nutrients and microorganisms could pass freely. The 1 cm-germinated rice seeds were placed in the bag with one seedling per pot; hence, the central-rooted and the outside non-rooted compartments contained the rhizosphere and the rhizosphere-extended soils, respectively. A set of mesocosm pots were incubated without rice plants, and considered as bulk soil. The selected rice varieties were grown for 90 days, wherein each treatment (five rice varieties and one bulk soil) contained triplicate pots for the four time points (at 0, 30, 60 and 90 days). The mesocosms were set up in a greenhouse with 65% humidity, a temperature regime of 35°C/28°C, light/dark photoperiod of 14 h/10 h and light intensity of 400 μmol m^−2^ s^−1^ provided by high-pressure sodium lamps. The mesocosms were placed in large plastic containers (height, 25 cm) and flooded with water every 24 hours to mimick paddy settings. Inorganic N (NH_4_^+^-N and NO_3_^−^-N) was extracted with 2.5 M KCl by shaking (1 h, 200 rpm) before colorimetric determination [20].

**Molecular analyses**

DNA was extracted from 0.5 g soil using a phenol-chloroform extraction method [26]. Extracted DNA was cleaned with AMPure® XP magnetic beads (Invitrogen, USA), according to the manufacturer's instructions. DNA concentrations and quality were measured using a Nanodrop (ND-1000 Spectrophotometer, Labtech). The abundance of archaeal and bacterial ammonia monooxygenase subunit A (*amoA*) gene was determined by qPCR using the crenamoA23F/crenamoA616R [2] and *amoA*-1F/*amoA*-2R [3] primer pairs, respectively, using standards and cycling conditions as described previously [4]. The total microbial abundance was determined using 16S rRNA gene primer pair 515F–806R and cycling conditions as described previously [5-6]. Amplification efficiencies for 16S rRNA gene, archaeal and bacterial *amoA* qPCR assays were 98, 87 and 96%, respectively, with r^2^ values > 0.99. The 16S rRNA gene and *amoA* (AOA and AOB) gene abundance was adjusted for dry soil weight and converted to their natural log values for further analysis.

For MiSeq Illumina sequencing, archaeal *amoA* genes, bacterial *amoA* genes and 16S rRNA genes were amplified using primer pair cren*amoA*23F/cren*amoA*616R [2], *amoA*-1F/*amoA*-2R [3] and 515F–806R [5-6], respectively, containing additional specific MiSeq-tailed sequences, following manufacturers’ recommendations. Triplicate amplification was done using the KAPA HiFi HotStart ReadyMix (Kapa Biosystems) with thermal cycling conditions described previously [7]. The PCR-amplified sequences were cleaned using AMPure XP beads (Beckman Coulter), and PCR-indexing was performed using Nextera XT Index Kit according to the manufacturer’s protocol.

Raw reads were processed to ASVs using the nf-core/ampliseq pipeline with custom parameter sets. Primers were first removed using cutadapt [8] before using DADA2 [9] to perform quality control and process reads to find amplicon sequencing variants (ASVs). Briefly, raw reads were truncated to remove low-quality bases (AOA *amoA*: forward, 240bp and reverse, 170bp; AOB *amoA*: forward, 280bp and reverse, 231bp; 16S rRNA gene: forward, 200bp and reverse, 150bp). Reads with more than 2 expected errors were filtered out. The core DADA2 algorithm was applied to identify unique sequence variants and merge paired reads, As the AOA *amoA* amplicon is too long for overlapping, these reads were concatenated with a sequence of 10 “N” bases in the middle. Taxonomic assignment was performed with the DADA2 implementation of the naïve Bayesian classifier method and a minimum bootstrap value of 80. Taxonomy was assigned to ASVs using GreenGenes2 [10] for 16S rRNA gene, and custom databases created from highly resolved phylogenies of AOA *amoA* [11] and AOB *amoA* [7]. A custom BLAST script was used to align ASVs to the target gene. Sequences with no hit, using an e-value threshold of 10, were discarded. For the *amoA* amplicons, ASVs were translated to protein sequences and any ASVs containing stop codons were removed from the analysis.

**Metabolomics**

The collected and filtered root exudates were evaporated until dryness using a rotary evaporator (Buchi Rotary Evaporator R-114 Series) at 40°C. The residues remaining in the round-bottom flask were dissolved in a series of 10 ml 90%, 5 ml 70% and 1 ml 50% HPLC grade methanol as a desalting approach and re-evaporated to dryness in a glass universal under N_2_. The dried exudates were then redissolved in 5 ml 90% HPLC grade methanol and re-evaporated to dryness in a 5 ml Eppendorf tube under N_2_. The dried exudates were then redissolved in 1.5 ml 80% HPLC grade methanol and re-evaporated to dryness in a 1.5 ml Eppendorf tube under N_2_. The collected dry root exudate mass was weighed and stored at -20 °C.

Chemical characterisation of the root exudates was performed by LC-MS analysis, using 0.15 mg of the dried root extracts re-dissolved into 250 µL 50% HPLC grade methanol. LC-MS analysis was carried out using the Bruker MAXIS II mass spectrometer (Bruker Corporation, Billerica, MA, USA) equipped with a Quadrupole-Time-of-Flight mass analyser joined to an HPLC (Agilent 1290 Infinity equipped with a diode array detector) (Agilent Technologies, Santa Clara, CA, USA) on a Kinetex EVO l C18 column (2.6 μm, 100 A, 2.1 × 100 mm) was used to analyse the sample. A mobile phase gradient of 0.1% formic acid in LC-MS-grade H_2_O:acetonitrile from 5 to 95% in 10 min, and isocratic elution at 95% for 2 min at a flow rate of 1 μL/min with MS acquisition for 12 min were employed in the UPLC-MS system. Mass spectra were measured in positive ion mode electrospray ionisation.

Raw Bruker files were converted to mzML format using Proteowizard MSConvert [12] with msLevel set to 1, polarity set to positive, and the peakPicking option selected using the ‘Vendor’ algorithm to convert profile data to centroid. Raw mzML files were converted to a feature table with a custom script using PyOpenMS v3.3.0 [13] implemented in Python v3.13.7. Mass trace detection used a noise threshold set to three times the mean signal intensity of blank injections (methanol only) and a mass accuracy of 2 ppm. During elution peak detection, peaks with full width at half maximum height outside the range of 1 - 60s were removed by setting the width_filtering parameter to “fixed”. During feature detection, mass traces without satellite isotopic traces were removed by setting the remove_single_traces parameter to “true”, and any features that fell two times the median absolute deviation below the median were removed to filter out low-quality features. During feature alignment, the error level for matching features between feature maps was set to 5 ppm, with the max_num_peaks_considered set to -1 (infinite). All other parameters were left at default values, and the feature table was exported in CSV format for further analysis.

The feature table was further processed in a Jupyter v1.1.1 notebook environment with Python v3.13.7. Data manipulation was performed with Pandas v2.3.2 and Numpy v2.3.3. Plotting was performed using Seaborn v0.13.2 and Matplotlib v3.10.6. Multivariate data analysis libraries specified below used R v4.4.3 through rpy2 v3.6.4. Features were removed that did not appear in all three replicates of any genotype, leaving 881 features. Median normalisation [14] was applied per ion within each analytical batch to correct for instrumental drift; where median values were zero, the median of non-zero values was used. Data were log_2_-transformed following the addition of a pseudocount (0.001). Euclidean distances between samples were calculated using vegdist from the vegan v2.7.1 R package with default parameters. The pcoa function from the ape v5.8.1 R package was used to perform Principal Coordinate Analysis (PCoA) on the precomputed Euclidean distances, without correction. Confidence ellipses for ordination were drawn using a custom Python function (<https://gist.github.com/CarstenSchelp/b992645537660bda692f218b562d0712>). Permutational Analysis of Variance (PERMANOVA) was performed using the adonis2() function from vegan v2.7.1 with 999 permutations and genotype as the explanatory variable. Pairwise comparisons between genotypes were performed using the pairwise.adonis() function from the pairwiseAdonis v0.4 R package with 999 permutations and Benjamini-Hochberg correction for multiple comparisons. Beta dispersion was calculated using the betadisper() function from vegan v2.7.1. The shapiro.test() function was used to test for data normality within groups, and the kruskal.test() function was used to test for differences in beta dispersion between genotypes in base R.

**Testing the BNI efficiency of root exudates and BNI *N*-butyldodecane-1-amine in cultures**

Three AOA and three AOB pure strains were selected to test the nitrifier inhibitory activity of both the root exudates and the identified chemical BNI compound. The selection included the AOA strains *Nitrososphaera viennensis* (EN76), isolated from an Austrian pH 8 garden soil [15], *Nitrosotalea sinensis* (Nd2), isolated from a Chinese pH 4.7 paddy soil [16], and *Candidatus* Nitrosocosmicus franklandianus (Nfr-C13), isolated from a Scottish pH 7.5 sandy loam soil [17], and the AOB strains, *Nitrosospira tenuis* NV12*,* isolated from Hawaiian soil [18], *Nitrosomonas europaea* ATCC 19718 and *Nitrosospira multiformis* ATCC 25196, obtained from NCIMB (<http://www.ncimb.com/>). The three AOA strains were grown in their respective modified freshwater medium as described previously for *N. viennensis, Ca.* N. franklandianus and *N. sinensis*, at 35°C, 40°C and 35°C, respectively. All three AOB strains, *N. tenuis* NV12, *N. europaea* ATCC 19718 and *N. multiformis* ATCC 25196 were grown using the modified Skinner and Walker medium at 28°C.

For each crude root exudate, 100 mg of dried exudates was initially dissolved in 1 ml of 50% filtered dimethyl sulfoxide (DMSO), and 10 µl of the dissolved root exudates were added to 5 ml of culture media to obtain a final concentration of 0.1% DMSO in culture media. This means a final dose of 1 mg of root exudate was tested for each culture. The DMSO concentration was chosen to reduce any inhibitory effect caused by DMSO on the tested nitrifier strains [19]. To remove any inhibition bias caused by trap solution salts or leftover soil phenolics, the dried control trap solution was also dissolved in 50% filtered DMSO and processed similarly to the root exudates.

The identified BNI compound, *N*-butyldodecane-1-amine (NBDA), was obtained from Sigma-Aldrich (purity of 96%). NBDA stock solutions were prepared using filtered 100% DMSO, and 5 µl of stock solution was added to a 5 ml medium at concentrations in the range of 0.2 - 50 μM, as required. NBDA mode of ammonia oxidation enzyme inhibition was analysed by incubating the AOA and AOB strains at 1 μM and 15 μM of NBDA, respectively, in the presence or absence of 3.25 mM hydroxylamine [20]. In addition, the inhibitory effect of NBDA was tested on three non-ammonia oxidiser strains grown in LB media at 35°C (*Escherichia coli* MG1655) or at 25°C (*Pseudomonas fluorescens* NCIMB 10586 and *Pseudomonas putida* PG NCIMB 9816). All the treatments using root extracts and the pure compound were carried out in quadruplicate.

Growth analyses and determination of inhibitory concentration at 80% inhibition (IC_80_) were performed as described previously [19]. Significant differences in (a) the inhibition between the different strains by root exudates, (b) the inhibition between AOA and AOB by root exudates, (c) the IC_80_ of NBDA between the different strains, (d) the nitrfier growth rates with NBDA supplemented in the presence and absence of hydroxylamine and (e) the growth rates of non-nitrifier strains in the presence or absence of NBDA were measured using multi-factorial linear modelling and a two-way ANOVA followed by post hoc tests using Tukey HSD and emmeans in R V4.1.2.

**N-butyldodecane-1-amine effect on nitrification dynamics in soil**

Microcosms, containing NBDA at 0, 100, 500, 1000 and 1500 µg NBDA kg^-1^ dry soil, were incubated for 7 days, during which N₂O emissions were measured daily using an Aeris analyser coupled to closed chambers. At the end of the incubation, soils were destructively sampled for qPCR quantification of AOA, AOB and bacterial 16S rRNA genes, and for determination of nitrate and ammonium concentrations. Inorganic N (NH_4_^+^-N and NO_3_^−^-N) was extracted with 2.5 M KCl by shaking (1 h, 200 rpm) before colourimetric determination [20]. The effects of NBDA dosage on N_2_O emissions, soil nitrifier abundance, and soil nitrogen dynamics were analysed using two-way ANOVA followed by post hoc tests using Tukey HSD and emmeans in R V4.3.1.

**Supplementary Figures:**

**Figure S1. Total microbial abundance in soil.** 16S rRNA gene abundance in bulk soil at day 0 and day 90 and in rhizosphere soil at day 90 (top panel), and inhibition of 16S rRNA gene abundance relative to bulk soil at day 90 (bottom panel), across five rice genotypes (Lemont, Koshihikari, IR64, Moroberekan and Azucena) and soil compartments (rhizosphere and extended rhizosphere), quantified by qPCR. Data are represented as bar plots, and standard errors (n = 3) are indicated by error bars. Different letters on top of each box denote significant differences (*P* < 0.05) in gene abundances, as determined using ANOVA and post-hoc analyses (Statistic S1).

**Figure S2. Decline in nitrifier abundance in bulk soil.** Reduction in AOA and AOB abundance in bulk soil over time. Data are presented as barplot and standard errors (n = 3) are indicated by error bars. Capital letters indicate significant differences among AOA treatments, whereas lowercase letters indicate significant differences among AOB treatments. Different letters denote statistically significant differences (*P* < 0.05) based on ANOVA followed by post hoc analyses (Statistic S2).

**Figure S3. Abundance of nitrifiers in soil.** The temporal changes in natural log-transformed ammonia monooxygenase (*amoA*) gene abundance (AOA or AOB) in the bulk, rhizosphere extended and rhizosphere soil compartments in the five rice genotypes (Lemont, Koshihikari, IR64, Moroberekan and Azucena) as determined by qPCR. Data are represented as bar plots, and standard errors (n = 3) are indicated by error bars.

**Figure S4. The ratio of abundance of nitrifiers in the soil compartments over time.** The ratio of ammonia oxidiser (AOA and AOB) abundance in the rhizosphere and extended rhizosphere soil compartments over time for the five rice genotypes investigated. The ratio of abundance was calculated as: nitrifier abundance in the rhizosphere or extended rhizosphere divided by nitrifier abundance in bulk soil. Data are represented as a boxplot (n = 3), and ns and *** on top of each boxplot pair denote significant differences between rhizosphere and extended rhizosphere ammonia oxidiser abundance with a *P* value > 0.05 and a *P* value < 0.001, respectively, tested using ANOVA (two-way interaction between time and soil compartments) (Statistic S4).

**Figure S5. The abundance of nitrifiers in the bulk soil.** The natural log-transformed ammonia-oxidiser (AOA and AOB) abundance inside and outside the mesh bag in the bulk soil pots over time. Data are represented as a boxplot (n = 3), with no significant differences (represented as ns with a *P* value > 0.05) in ammonia oxidiser (AOA and AOB) abundance between outside and inside mesh bag at each time point, analysed using ANOVA (two-way interaction between time and soil compartmentalisation (inside and outside the mesh bag)).

**Figure S6. Biological nitrification inhibition of nitrifiers in culture by rice root exudates.** Growth inhibition of ammonia oxidisers (AOA and AOB) in culture using an equal mass of root exudates from the five rice genotypes (Lemont, Koshihikari, IR64, Moroberekan and Azucena). Different letters on top of each bar denote significant differences (*P* value < 0.01) in the nitrifier inhibition between the different rice genotypes, tested using ANOVA (strain effect), within each rice genotype. Strains used in the study were AOB – *N. europaea, N. multiformis* and *N. tenuis* and AOA – *N. viennensis*, *N. sinensis* and *Ca.* N. franklandianus. Data are presented as barplot and standard errors (n = 4) are indicated by error bars (Statistic S7).

**Figure S7. Soil ammonium and nitrate dynamics in rice rhizosphere over time.** Soil ammonium (NH_4_^+^–N; top) and nitrate (NO_3_^–^–N; bottom) concentrations measured over 90 days in bulk soil and rhizosphere soil of the five rice genotypes (Lemont, Koshihikari, IR64, Moroberekan and Azucena). Values are expressed as µg N per g of dry soil. No nitrite was observed during measurements. Data are presented as barplot and standard errors (n = 3) are indicated by error bars.

**Figure S8.** **Sequencing depth.** Sequence read numbers for the (left to right) AOB *amoA*, AOA *amoA* and 16S rRNA in the five rice genotypes (Lemont, Koshihikari, IR64, Moroberekan and Azucena) and within all soil compartments (bulk, extended rhizosphere and rhizosphere) at the start (day 0) and end (day 90) of the experiment. Data are represented as dot plot (n = 3).

**Figure S9. Rarefaction curves.** Rarefaction curves for the alpha diversity indices – Richness, Shannon and Inverse Simpson (from left to right) for AOB *amoA*, AOA *amoA* and 16S rRNA (from top to bottom) in the five rice genotypes (Lemont, Koshihikari, IR64, Moroberekan and Azucena) and within all soil compartments (bulk, extended rhizosphere and rhizosphere) at the start (day 0) and end (day 90) of experiment. Data are represented as dot and line plot (n = 3).

**Figure S10. Ammonia oxidiser community composition.** Community composition of AOB *amoA* (top) and AOA *amoA* (bottom) in the bulk soil and in the five rice genotypes (Lemont, Koshihikari, IR64, Moroberekan and Azucena) within the extended rhizosphere (ER) and rhizosphere (R) at the start (day 0) and end (day 90) of the experiment. For the AOB, all represented clusters fall within *Nitrosospira*. For AOA, the order levels represented are NS, which is Nitrososphaerales; NP, which is Nitrosopumilates; and NT, which is Nitrosotaleales. Data are represented as barplots (n = 3).

**Figure S11.** **Alpha diversity.** Alpha diversity indices of Richness (top row), Shannon (middle row) and Inverse Simpson (bottom row) for AOB *amoA*, AOA *amoA* and 16S rRNA (from left to right) in the bulk soil and in the five rice genotypes (Lemont, Koshihikari, IR64, Moroberekan and Azucena) within the extended rhizosphere and rhizosphere) at the start (day 0) and end (day 90) of the experiment. Data are represented as boxplot (n = 3). Different letters on top of each box denote significant differences (*P* value < 0.05) in each diversity index, tested using ANOVA (two-way interaction between, genotype and soil compartment), within each microbial marker gene (Statistic S8, S10 and S11).

**Figure S12. PCoA plot of beta diversity.** The principal coordinates analysis (PCoA) ordination plots representing the changes in the beta diversity of (from left to right) AOB *amoA*, AOA *amoA* and 16S rRNA analysed using Bray Curtis index for in the bulk soil and in the five rice genotypes (Lemont, Koshihikari, IR64, Moroberekan and Azucena) within the extended rhizosphere and the rhizosphere at the start (day 0) and end (day 90) of experiment. Each panel represents the significance of the variance explained by the model (*P* value < 5.5×10^-3^) determined using PERMANOVA (permutations = 9,999). Data are represented as dot plot (n = 3) (Statistic S8, S10 and S11).

**Figure S13.** **Conceptual correlation network.** Conceptual correlation network summarising relationships among plant physiological traits, BNI efficiency, root exudation metabolome, nitrifier diversity and general microbial diversity. (A) Relationships among plant physiological traits (shoot weight, root weight, shoot N content and exudate weight), BNI efficiency expressed as AOB and AOA decline rates (BNI.AOB and BNI.AOA), and root exudate metabolite profiles represented by PCoA axes 1 and 2. (B) Correlations among AOA, AOB and total bacterial (16S rRNA gene) diversity in the rhizosphere and extended-rhizosphere, using Inverse Simpson diversity for α-diversity and PCoA1 for β-diversity. Correlations linking plant physiology, BNI efficiency and root exudation with (C) AOB, (D) AOA and (E) total bacterial diversity in the rhizosphere. Black arrows connecting modules represent significant Mantel test correlations between data groups, whereas grey arrows show tested but non-significant correlations. Inset correlation matrices adjacent to each connection show pairwise Pearson correlations among variables within interacting modules. Blue indicates positive correlations and red indicates negative correlations, with ellipse shape and colour intensity proportional to correlation strength. Symbols *, ** and *** denote statistical significance at *P* < 0.05, *P* < 0.01 and *P* < 0.001, respectively. Non-significant interactions were removed from the correlation plots.

**Figure S14. Microbial community composition plots.** Community composition of 16S rRNA gene at phylum, class and order level in the bulk soil and in the five rice genotypes (Lemont, Koshihikari, IR64, Moroberekan and Azucena) within the extended rhizosphere (ER) and the rhizosphere (R) at the start (day 0) and end (day 90) of the experiment. Data are represented as bar plot (n = 3).

**Figure S15: Correlation between plant traits and BNI efficiency.** A. Root and Shoot biomass of the five rice genotypes (Lemont, Koshihikari, IR64, Moroberekan and Azucena). Data are presented as a bar plot, and standard errors (n = 3) are indicated by error bars. Different letters on top of each bar denote significant differences (*P* value < 0.05), tested using ANOVA. B. Pearson correlation matrix showing relationships among plant traits, nitrogen uptake, root exudation and BNI efficiency. Blue indicates positive correlations and red indicates negative correlations, with ellipse shape and colour intensity corresponding to correlation strength. Symbols *, **, *** and ns denote statistical significance at *P* < 0.05, *P* < 0.01, *P* < 0.001 and *P* > 0.05, respectively (Statistic S12).

**Figure S16: Density distribution of mass-to-charge (m/z) values detected in the LC-MS analysis of all the rice root exudates.**

**Figure S17: PCoA of rice metabolome.** Two-dimensional principal coordinate analysis (PCoA) showing ordination across 3 axes of high-molecular-weight root exudate composition from 90-day-old rice plants, based on LC–MS profiles (n = 3 per genotype).

**Figure S18. Identification of rice BNI,** **N-butyldodecan-1-amine (NBDA).** Panel A. The LC-MS chromatogram region of the identified compound NBDA showing the fragmentation pattern in the positive ESI-MS mode (UV detection at 228 nm) in the Lemont root exudates. Panel B. The proposed molecular structures of the fragments observed in the LC-MS chromatogram. Panel C. The molecular structure of the identified compound NBDA.

**Figure S19: Relative quantification of NBDA in rice root exudates by LC–MS.** NBDA signal of peak intensity in day-90 exudates across the five rice genotypes. Different letters on top of each bar denote significant differences (*P* value < 0.05), tested using ANOVA. Data are presented as a bar plot, and standard errors (n = 3) are indicated by error bars. Azucena showed no peak for NBDA.

**Figure S20. Inhibition mechanism of rice BNI, NBDA.** Comparison of the growth rates of five ammonia-oxidising strains with and without hydroxylamine supplementation in the presence and absence of NBDA. Nitrifier strains used in the study are AOB – *N. europaea* and *N. multiformis,* AOA – *N. viennensis*, *N. sinensis* and *Ca.* N. franklandianus. Data are represented as bar plots, standard errors (n = 4) are indicated by error bars, and different letters within each subplot denote significant differences (*P* value < 0.05) in inhibition based on ANOVA (growth condition effect), tested separately for each strain (Statistic S13).

**Figure S21. Inhibitory effects of NBDA on non-nitrifying microorganisms.** Comparison of growth rates of three non-nitrifier strains, *Escherichia coli* MG1655, *Pseudomonas fluorescens* NCIMB 10586 and *Pseudomonas putida* PG NCIMB 9816, in the presence of different concentrations of NBDA. Different letters within each subplot denote significant differences (*P* value < 0.05) in inhibition for each strain based on ANOVA and Tukey post hoc test. All data are presented as bar plots, and standard errors (n=4) are indicated by error bars (Statistic S13).

**Figure S22. Inhibitory effects of NBDA dosage on soil nitrification dynamics.** A. Daily N_2_O fluxes under increasing NBDA concentrations (0, 100, 500, 1000 and 1500 µg g^–1^ soil). B. Reduction in cumulative N_2_O emissions relative to the control across NBDA treatments. C. Soil N derived from nitrate at 7^th^ day across NBDA concentrations. All data are presented as bar plots, and standard errors (n=3) are indicated by error bars. Different letters within each subplot denote significant differences (*P* value < 0.05) based on ANOVA followed by post hoc analyses (Statistic S14).

**Figure S23. Inhibitory effects of NBDA dosage on soil microbial dynamics.** A. Inhibition of AOB abundance after 7 days of NBDA treatment at 0, 100, 500, 1000 and 1500 µg g^–1^ soil. Changes in B. AOB and C. 16S rRNA gene abundance after 7 day incubation across NBDA concentrations. All data are presented as bar plots, and standard errors (n=3) are indicated by error bars. Different letters within each subplot denote significant differences (*P* value < 0.05) based on ANOVA followed by post hoc analyses (Statistic S14).

**Supplementary Tables**

**Table S1. Reported inhibitory concentrations of BNI compounds against nitrifiers.** The table summarises the BNI compound class, compound name, plant source, and mean inhibitory concentrations causing either 80% or 50% inhibition (IC₈₀ or IC₅₀; μM) of ammonia-oxidising archaea (AOA) and ammonia-oxidising bacteria (AOB), as determined using activity, growth, or bioluminescence assays. Where multiple test strains were used, inhibitory concentrations are reported as mean ± SD.

| **Compound class** | **Plant source** | **BNI compound** | **AOA mean IC₈₀ (µM)** | **AOB mean IC₈₀ (µM)** | **Inhibition assay** | **Reference** |
| --- | --- | --- | --- | --- | --- | --- |
| Amine | Rice | NBDA | 4 ± 1 | 38 ± 13 | Growth assay | This study |
| Fatty alcohol | Rice | 1,9-Decanediol | 377 ± 269 | 793 ± 500 | Growth assay | [19] |
| Phenolic acids | Rice | Syringic acid | – | >2500 | Activity assay | [22] |
| Phenolic acids | Rice | Ferulic acid | – | >2500 | Activity assay | [22] |
| Phenolic acids | Rice | Shikimic acid | – | >2500 | Activity assay | [22] |
| Phenolic acids | Sorghum | MHPP | 667 ± 405 | 287 ± 214 | Growth assay | [19] |
| Phenolic acids | Sorghum | Sorgoleone | – | 13 | Bioluminescence | [21] |
| Flavonoid | Sorghum | Sakuranetin | 119 ± 77 | 354 ± 255 | Growth assay | [19] |
| Fatty acid derivatives | *B. humidicola* | Linoleic acid | 15 ± 19 | 510 ± 201 | Growth assay | [19] |
| Fatty acid derivatives | *B. humidicola* | Linolenic acid | 11 ± 9 | 259 ± 79 | Growth assay | [19] |
| Fatty acid derivatives | *B. humidicola* | Methyl linoleate | 18 ± 6 | 832 ± 259 | Growth assay | [19] |
| Terpene | *B. humidicola* | Brachialactone | – | 10.6 | Bioluminescence | [23] |
| Benzoxazinoids | Maize | Zeanone | – | 24 ± 12 | Bioluminescence | [24] |
| Benzoxazinoids | Maize | HDMBOA | – | 70 | Bioluminescence | [25] |
| Benzoxazinoids | Maize | HMBOA | – | 100 | Bioluminescence | [25] |
| Benzoxazinoids | Maize | HMBOA-β-glucoside | – | 200 | Bioluminescence | [25] |
| Amino acid | Kernza | Phenylalanine | – | ~50 | Growth assay¹ | [26] |
|  |  |  | IC5₀ (µM) | IC5₀ (µM) |  |  |
| Phenolic acids | *B. humidicola* | Ethyl-*p*-coumarate | – | 16.0 ± 0.5 | Bioluminescence | [27] |
| Phenolic acids | *B. humidicola* | Propyl-*p*-coumarate | – | 16.0 ± 1.0 | Bioluminescence | [27] |
| Phenolic acids | *B. humidicola* | Butyl-*p*-coumarate | – | 36.0 ± 1.5 | Bioluminescence | [27] |
| Phenolic acids | *B. humidicola* | Methyl ferulate | – | 4.4 ± 0.1 | Bioluminescence | [27] |
| Phenolic acids | *B. humidicola* | Ethyl ferulate | – | 0.2 ± 0.1 | Bioluminescence | [27] |
| Phenolic acids | *B. humidicola* | Propyl ferulate | – | 2.2 ± 0.10 | Bioluminescence | [27] |
| Phenolic acids | *B. humidicola* | Butyl ferulate | – | 4.2 ± 0.1 | Bioluminescence | [27] |
| Phenolic acids | – | Caffeic acid | 19.5 ± 6.0 | 171.5 ± 16.2 | Growth assay | [28] |
| Phenolic acids | – | Chlorogenic acid | 429.4 ± 246.2 | 107.2 ± 29.2 | Growth assay | [28] |
| Phenolic acids | – | Quinic acid | 1596.6 ± 124.1 | >300 | Growth assay | [28] |

**Supplementary References:**

1. Kemp JS, Paterson E, Gammack SM. et al. Leaching of genetically modified *Pseudomonas fluorescens* through organic soils: influence of temperature, soil pH, and roots. *Biol Fertil Soils* 1992;13(4):218–24. <https://doi.org/10.1007/BF00340579>
2. Tourna M, Freitag TE, Nicol GW. et al. Growth, activity and temperature responses of AOA and AOB in soil microcosms. *Environ Microbiol* 2008;10(5):1357–64. <https://doi.org/10.1111/j.1462-2920.2007.01563.x>
3. Rotthauwe JH, Witzel KP, Liesack W. The amoA gene as a functional marker of ammonia-oxidising populations. *Appl Environ Microbiol* 1997;63(12):4704–12. <https://doi.org/10.1128/aem.63.12.4704-4712.1997>
4. Thion C, Prosser JI. Differential response of non-adapted AOA and AOB to drying–rewetting stress. *FEMS Microbiol Ecol* 2014;90(2):380–9. 10.1111/1574-6941.12395
5. Apprill A, McNally S, Parsons R. et al. Minor revision to V4 SSU rRNA 806R primer greatly increases detection of SAR11. *Aquat Microb Ecol* 2015;75(2):129–37. <https://doi.org/10.3354/ame01753>
6. Caporaso JG, Lauber CL, Walters WA. et al. Global patterns of 16S rRNA diversity at a depth of millions of sequences per sample. *Proc Natl Acad* Sci USA 2011;108(Suppl 1):4516–22. [10.1073/pnas.1000080107](https://doi.org/10.1073/pnas.1000080107)
7. Aigle A, Prosser JI, Gubry-Rangin C. The application of high-throughput sequencing technology to analysis of *amoA* phylogeny and environmental niche specialisation of terrestrial bacterial ammonia-oxidisers. *Environ Microbiome* 2019;14(1).  <https://doi.org/10.1186/s40793-019-0342-6>
8. Martin M. Cutadapt removes adapter sequences from high-throughput sequencing reads. *EMBnet J* 2011;17(1):10–12. <https://doi.org/10.14806/ej.17.1.200>
9. Callahan BJ, McMurdie PJ, Rosen MJ. et al. DADA2: high-resolution sample inference from Illumina amplicon data. *Nat Methods* 2016;13(7):581–3. [10.1038/nmeth.3869](https://doi.org/10.1038/nmeth.3869)
10. McDonald D, Jiang Y, Balaban M. et al. Greengenes2 unifies microbial data in a single reference tree. *Nat Biotechnol* 2024;42:715–8.  <https://doi.org/10.1038/s41587-023-01845-1>
11. Alves RJ, Minh BQ, Urich T. et al. Unifying the global phylogeny and environmental distribution of ammonia-oxidising archaea based on *amoA* genes. *Nat Commun* 2018;9, 1517. <https://doi.org/10.1038/s41467-018-03861-1>
12. Chambers MC, MacLean B, Burke R. et al. A cross-platform toolkit for mass spectrometry and proteomics. *Nat Biotechnol* 2012; 30, 918–920. <https://doi.org/10.1038/nbt.2377>
13. Röst HL, Schmitt U, Aebersold R. et al. pyOpenMS: a Python-based interface to the OpenMS mass-spectrometry algorithm library. *Proteomics* 2014;14(1):74-77. <https://doi.org/10.1002/pmic.201300246>
14. Wulff J.E. and Mitchell M.W. A Comparison of Various Normalization Methods for LC/MS Metabolomics Data, *Adv. Biosci. Biotechnol.*, 2018; 9 : 339–351. <https://doi.org/10.4236/abb.2018.98022>
15. Tourna M, Stieglmeier M, Spang A. et al. *Nitrososphaera viennensis*, an ammonia oxidizing archaeon from soil. *Proc Natl Acad Sci USA* 2011;108(20):8420–5. <https://doi.org/10.1073/pnas.1013488108>
16. Lehtovirta-Morley LE, Ge C, Ross J. et al. Characterisation of terrestrial acidophilic archaeal ammonia oxidisers and their inhibition and stimulation by organic compounds. *FEMS Microbiol Ecol* 2014;89(3):542–52. <https://doi.org/10.1111/1574-6941.12353>
17. Lehtovirta-Morley LE, Ross J, Hink L. et al. Isolation of ‘*Candidatus* Nitrosocosmicus franklandus’, a novel ureolytic soil archaeal ammonia oxidiser with tolerance to high ammonia concentration. *FEMS Microbiol Ecol* 2016;92(5):fiw057. <https://doi.org/10.1093/femsec/fiw057>
18. Harms H, Koops HP, Wehrmann H. An ammonia-oxidizing bacterium, *Nitrosovibrio tenuis* nov. gen. nov. sp. *Arch Microbiol* 1976;108(1):105–11. [10.1007/BF00425099](https://doi.org/10.1007/bf00425099)
19. Kaur-Bhambra J, Wardak DLR, Prosser JI. et al. Revisiting plant BNI efficiency using multiple archaeal and bacterial ammonia-oxidiser cultures. *Biol Fertil Soils* 2021;58(3):241–9. <https://doi.org/10.1007/s00374-020-01533-1>
20. Hink L, Gubry-Rangin C, Nicol GW. et al. The consequences of niche and physiological differentiation of archaeal and bacterial ammonia oxidisers for nitrous oxide emissions. *ISME J* 2018;12(4):1084–93. <https://doi.org/10.1038/s41396-017-0025-5>
21. Subbarao GV, Nakahara K, Ishikawa T. et al. Biological nitrification inhibition (BNI) activity in sorghum and its characterization. *Plant Soil* 2013;366(1–2):243–59. <https://doi.org/10.1007/s11104-012-1419-9>
22. Lu Y, Zhang X, Ma M. et al. Syringic acid from rice as a BNI and urease inhibitor; synergism with 1,9-decanediol. *Biol Fertil Soils* 2021;58(3):277–89. <https://doi.org/10.1007/s00374-021-01584-y>
23. Egenolf K, Conrad J, Schöne J. et al. Brachialactone isomers and derivatives of *Brachiaria humidicola* reveal contrasting nitrification-inhibiting activity. *Plant Physiol Biochem* 2020;154:491–7. <https://doi.org/10.1016/j.plaphy.2020.06.004>
24. Otaka J, Subbarao GV, Ono H. Biological nitrification inhibition in maize—hydrophobic inhibitors from root exudates. *Biol Fertil Soils* 2021;58(3). <https://doi.org/10.1007/s00374-021-01577-x>
25. Otaka J, Subbarao GV, Jiang M. et al. Isolation and characterisation of hydrophilic BNI 6-methoxy-2(3H)-benzoxazolone (MBOA) from maize roots. *Plant Soil* 2023;489(1):341–59. [10.1007/s11104-023-06021-7](https://doi.org/10.1007/s11104-023-06021-7?urlappend=%3Futm_source%3Dresearchgate.net%26utm_medium%3Darticle)
26. Issifu S, Acharya P, Schöne J. et al. Metabolome fingerprinting reveals multiple nitrification inhibitors in *Thinopyrum intermedium*. *Plant-Environ Interact* 2024;5(5). <https://doi.org/10.1002/pei3.70012>
27. Gopalakrishnan S, Subbarao GV, Nakahara K. et al. Nitrification inhibitors from the root tissues of *Brachiaria humidicola*, a tropical grass. *J Agric Food Chem* 2007;55(4):1385–90. [10.1021/jf062593o](https://doi.org/10.1021/jf062593o)
28. Kolovou M, Panagiotou D, Süße L. et al. Assessing the activity of different plant-derived molecules and potential biological nitrification inhibitors on a range of soil ammonia- and nitrite-oxidizing strains. *Appl Environ Microbiol* 2023;89(11). <https://doi.org/10.1128/aem.01380-23>
